# Recurrent Episodes of Dissociative Fugue with Comorbid Severe Depression and Alcohol Dependence Syndrome

**DOI:** 10.1155/2022/7362823

**Published:** 2022-09-05

**Authors:** Dennis Bomansang Daliri, Agani Afaya, William H. F. Koomson, Emmanuel Akatibo

**Affiliations:** ^1^Presbyterian Regional Mental Health Center, Bolgatanga, Ghana; ^2^Mo-Im Kim Nursing Research Institute, College of Nursing, Yonsei University, Republic of Korea; ^3^Department of Nursing, School of Nursing and Midwifery, University of Health and Allied Sciences, Ho, Ghana; ^4^Department of Psychiatry, School of Medicine and Health Sciences, University for Development Studies, Tamale, Ghana; ^5^Department of Internal Medicine, Upper East Regional Hospital, Bolgatanga, Ghana

## Abstract

The rarity and close resemblance to other mental health conditions of dissociative fugue make it difficult to diagnose. Akin to a culture-bound syndrome, most African countries have their local explanation for this unique presentation and therefore people may not seek evidence-based health care but rather may resort to faith-based-treatment which may not give the best results. This is the case of a 39-year-old man who has experienced about five episodes of dissociative fugue over nine years. This case points out the comorbidities associated with the condition. We report this case to increase awareness of the condition and to bring to the fore the need for further studies into psychopathology and the treatment modalities.

## 1. Introduction

According to the International Classification of Diseases 10^th^ Edition, a dissociative fugue is a condition that is characterized by sudden purposeful travel away from one's place of residence or work, complete or partial amnesia of one's past and identity, causing the individual to assume a different identity [1]. It has been described by Rajah et al. as “a protective activation of altered states of consciousness in reaction to overwhelming psychological trauma” [2].

Dissociative fugue is usually preceded by a stressful event such as bereavement, trauma, loss of employment, abuses, and disasters. The condition usually is said to last from a few days to months, and the patient may look normal to independent observers, especially when a different identity is assumed.

The global prevalence of dissociative fugue has been estimated to be 0.2% [3]. There is however no country-specific prevalence for Ghana. It is said to be more common among adults as compared to children [4] and is often associated with other conditions such as posttraumatic stress disorder, depression, anxiety, and substance abuse [5].

In many African countries including Ghana, it is believed that people may suddenly be abducted by invisible beings said to be dwarfs, described as being extremely short with peculiar features. The abductees are alleged to be hidden by their captors in the forest for several days to years after which they return with some magical powers [6, 7]. This belief may be an attempt to describe dissociative fugue.

There is no specific medication for the treatment of dissociative fugue. Treatment involves helping the patient to deal with or come to terms with the social or environmental stressors that triggered the fugue through psychotherapy [4] and comorbid conditions appropriately treated. We report a case of dissociative fugue with comorbid severe depression with suicidality and alcohol dependence syndrome.

## 2. Case Report

We report a case of Mr. A.J., a 39-year-old male who resides in a suburb of the Upper East Region of Ghana in West Africa. He is a university graduate with second-class upper honors in mechanical engineering. He was reported missing from home for two weeks until his elder sister received a call from a hospital (which is about 93 km away from the patient's place of residence) that he had been brought in for medical attention by a Good Samaritan who found the patient in the community looking unkempt and exhausted. The patient was noted to have no recollection of his identity, where he was or how he got to the said location. He was then taken to the health facility in the community for medical care. The patient could however remember his sister's phone number, and hence, she was contacted.

On the day of his alleged disappearance from home, Mr. A.J. was last reading a novel while seated in front of his home. He had lost his job a few days prior and was very worried. He did not remember how and when he left home to the unknown destination.

The relatives said that this was the patient's fourth experience of such an episode in a space of 9 years. The first episode occurred in 2013 while working in the southwestern part of Ghana, and he suddenly left his place of work and found himself in his hometown which is in the Upper East Region of Ghana, 877 km away from his place of abode. His family was surprised to see him because he did not inform them of his visit. He was fully conscious but could not remember where he was and had no memory of the journey or how he paid for the trip. This event kept him absent from work for five days, resulting in a dismissal from the job. Before this episode, he had been having symptoms of generalized anxiety disorder which made him unable to give his best at work.

The second episode was 7 years ago. Prior to this, the patient had lost his job and was enduring a lot of pressure from the family over the need to provide. He felt extremely overwhelmed and suffered sleepless nights. He is said to have left home and lived in a cemetery for two weeks. The patient was noted to have been unkempt and exhausted when he was found. He had no memory of what brought him there nor his identity although he was fully conscious. He was sent to a nearby hospital where he was admitted and given some medications. Two days after the admission, he regained memory of his identity and was wondering how he got to the hospital. He however did not remember what had happened within the two weeks of being away from home.

The third episode occurred 4 years ago after he had lost another job. He is reported to have left home for close to two months and was found to have been staying on a hill in a community, 30 km away from his home. He was unable to provide any information about himself. After about 8 weeks of staying in the community, he walked to a stranger to ask for the name of the community and how he got there. He was able to remember the contact of his elder sister and so the necessary arrangements were made for his safe return. A summary of the various events and their corresponding features is shown in Table 1.

The current episode has been followed by sadness, anhedonia, poor sleep, weight loss, and suicidality with three previous attempts. He has visited several medical facilities and faith-based treatment centers and has not observed any improvement in his condition. He was informed by three independent spiritual healers that his episodes are a result of “abduction by dwarfs,” and thus, nothing could be done about it. He therefore resorted to excessive alcohol consumption to help him to sleep at night, gradually leading to tolerance and dependence on alcohol. He however denied the use of other psychoactive substances.

A mental state examination of the patient revealed a well-dressed and kempt young man who was fully conscious. He had some scarifications on the left precordium and some weight loss evidenced by prominent zygomatic bones. He had a depressed mood, reactive affect, and abnormal speech. He had suicidal ideations but did not desire to carry out any act of self-harm or suicide. He was well oriented to time, place, and person. His attention and concentration were good. His short-term memory was intact, and he could not recollect any details of events that occurred during the recent two-week absence from home. He had good judgment, intact abstract thinking, and good insight into his condition.

The patient described his experience as very distressing and depressing because it has cost him his employment three times, and as a result, he is currently dependent on family members for his sustenance. He recounted that each time he recovered from an episode, he felt depressed considering the impact of the episode on him. Currently, the patient is worried about his inability to recollect where he dropped his phone and purse which contained all his identity documents during the recent episode.

Findings of the neurological and other physical examinations were normal.

The patient had a score of 4 on the Sad Persons Scale which has been reported as being appropriate for assessing suicidal attempts [8].

With regard to the Dissociation Experience Scale (DES), the patient scored 58 indicating a high likelihood of a dissociative disorder. This scale has been described as effective in screening for dissociative disorders [9].

Diagnoses are as follows: dissociative fugue disorder with comorbid severe depression with suicidality and alcohol dependence syndrome at the decision stage of change.

### 2.1. Investigation Results

The results of his blood investigations were unremarkable except for a marginally elevated gamma-glutamyl transferase (GGT).

An electroencephalogram (EEG) showed no evidence of seizures.

The patient however could not afford the cost of a brain Magnetic-Resonance Imaging (MRI) scan.

### 2.2. Management

Given the clinical evidence with the investigations and confirmed diagnosis, the patient was put on close observation against suicide and the possibility of leaving home or traveling. He was managed on outpatient basis as there was no in-patient mental health facility for admission. Daily visits to the clinic were scheduled during which suicidal intent assessment was conducted and appropriate action taken consequently.

His depression was medically managed with Citalopram tablets, and a program for medically assisted alcohol withdrawal was initiated on outpatient basis. He was also referred to the clinical psychologist for psychotherapy sessions.

Three months after the initiation of treatment, the patient has had no episode of dissociative fugue. He no longer has suicidal ideations but has mild depressive symptoms. He has managed to completely abstain from alcohol.

Mental state examination revealed he was still unable to remember events from the last episode and still wondering where he left his mobile phone and identity cards.

## 3. Discussion

Dissociative fugue may be difficult to recognize and hence underdiagnosed [10] because of its rare nature and its close resemblance to other conditions [11]. It may be confused with many other conditions such as epilepsy, dementia, and other dissociative disorders, schizophrenia, and bipolar affective disorder (manic episode) [5, 12], and hence, a thorough inquiry and investigations are required to rule it out or confirm it.

The cultural setting and beliefs about this condition may cause many to remain untreated as the origin of symptoms may be attributed to spiritual causes such as a spiritual abduction or “abduction by dwarfs” as observed in the above case [6, 7]. The persistence of the condition may be a result of a lack of proper assessment and diagnosis. The condition may result in certain consequences such as causing distress, severing relationships, and also affecting occupations or employments [13]. Evidence supporting a biological basis for dissociative disorders, in general, is inconsistent with the more consistent linkage to posttraumatic stress disorder [14]. It is also known that alcohol dependence is associated with dissociative disorders. People who abuse drugs including alcohol may exhibit symptoms of dissociative disorders [15]; however, in the case in point, the dissociative fugue preceded the abuse of alcohol.

## 4. Strengths and Limitations

This is a case of a dissociative fugue with comorbid alcohol dependence syndrome. It gives a vivid account of the condition and its associated comorbidity. Occurring in an African setting, it provides some insight into the local beliefs of “abduction by dwarfs.” It also highlights the key features of the condition as presented in the literature. This report sets a good ground for the generation of hypotheses to further investigate this psychopathology. Despite these strengths, the diagnosis was made for the first time after the patient had recovered from this last episode. This account might suffer from recall bias. Also, as a feature of all case reports, it lacks generalizability and the ability to establish cause and effect.

## 5. Conclusion

Dissociative fugue is a rare clinical entity that can recur like any other dissociative disorder when the individual faces exceptional perceived stress. Due to the rarity of cases of dissociative fugue, more education will be required to enlighten physicians on the condition and emphasize the need for a multidisciplinary approach to its management, involving a physician, a therapist, and possibly a social worker.

## Figures and Tables

**Table 1:** The dates, stressful life events associated with, and features of each episode.

Year	Episode	Stressful life event	Features
2013	First	Symptoms of generalized anxiety affect his performance at work.	Suddenly traveled over 877 km from his place of work to his hometown with no memory of the journey lasting for 5 days
2015	Second	Loss of employment	Lived in a cemetery for 2 weeks and had no memory of his identity and how he got to that location
2018	Third	Loss of employment	Lived on top of a hill for 2 months and did not remember how he arrived at the destination. He had forgotten his identity
2022	Fourth	Unemployment	Traveled a one-and-a- half drive journey from his home to an unfamiliar location and lived there for 2 weeks with no memory of his identity

## Data Availability

For the purpose of maintaining confidentiality, data from this case report cannot be made readily available. It may however be made available upon a reasonable request from the Director of the Presbyterian Regional Mental Health Center on the following email address: info@prmhc.org.
